# Methylation of *PAX1* gene promoter in the prediction of concurrent chemo-radiotherapy efficacy in cervical cancer

**DOI:** 10.7150/jca.57460

**Published:** 2021-06-22

**Authors:** Xuanxuan Li, Xue. Zhou, Manting Zeng, Yangying Zhou, Yu Zhang, Yu-Ligh Liou, Hong Zhu

**Affiliations:** 1 Department of Oncology, Xiangya Hospital, Central South University, Hunan 410008 China.; 2 Department of Obstetrics and Gynecology, Xiangya Hospital, Central South University, Hunan 410008 China.; 3 Department of Clinical Pharmacology, Xiangya Hospital, Central South University, Hunan 410008 China.

**Keywords:** Cervical cancer, *PAX1*, DNA methylation, Current chemo-radiotherapy, Biomarkers

## Abstract

**Objectives:** Cervical cancer is the fourth leading cause of cancer death among women worldwide. In currently, aberrant methylation of *PAX1* is found in variety of solid tumors, including cervical cancer. In addition, the role of *PAX1* gene methylation in cervical cancer and precancerous lesions screening has been confirmed in previous study. Here, we evaluated the predictive value of *PAX1* methylation in concurrent chemo-radiotherapy (CCRT) outcomes in cervical cancer.

**Methods:** This study enrolled 82 cervical cancer patients from August 2018 to August 2020. We compared the clinical results between different *PAX1* methylation status. Hyper-methylation patients were subjects to MRI and quantitative methylation-specific PCR (QMSP) for* PAX1* before, in the middle, immediately after, 1 month and 3 months after CCRT. The changes in *PAX1* methylation during CCRT were analyzed.

**Results:** The lower *PAX1* methylation status were related to a poor tumor response. Based on the MRI findings three months post-treatment, the hypermethylated patients were classified into the complete response (CR; n=50) and partial remission (PR; n=18) groups. The average *PAX1* △Cp value of CR and PR groups before radiotherapy was 5.08±1.98 and 4.32±2.00 respectively, and after concurrent chemo-radiotherapy was significantly increased to 17.35±4.96 and 16.99±6.17, respectively (P<0.05). Furthermore, the *PAX1* △Cp value between CR and PR groups were significantly different at mid-treatment and performed well in predicting short-term efficacy (AUC 0.84) in this period, and its sensitivity and specificity for predicting PR were 0.72 and 0.88, respectively.

**Conclusion:** The *PAX1* methylation level may predict the sensitivity and efficacy of CCRT in cervical cancer.

## Introduction

Cervical cancer ranks fourth among gynecological malignancies in terms of both incidence and mortality rates, and 80% of the newly diagnosed cases are from developing countries 1. Radiotherapy is a common treatment modality for cervical cancer, and has significantly improved patient prognosis, prolonged survival rates, and reduced the risk of recurrence and distant metastasis. However, some patients are resistant to radiotherapy, and its side effects such as radiation enteritis and bone marrow suppression significantly reduce the quality of life. Therefore, early assessment of tumor response is critical to avoid exposing the potentially unresponsive patients to radiotoxicity. Currently, radiotherapeutic effects on cervical tumors are monitored by computed tomography (CT), magnetic resonance imaging (MRI) and other imaging techniques. However, they may take weeks or even months to ascertain treatment efficacy 2. Therefore, the high costs, long duration and increased potential radiation burden significantly limit the utility of MRI during clinical follow-up. On the other hand, the lag of tumor histological regression, oedema and inflammation of surrounding normal tissue caused by radiation changes may hide the morphological changes of tumor to a certain extent, which may lead to deviation in evaluating tumor measurement 3. In contrast, molecular biomarkers can enable real-time monitoring and early prediction of treatment efficacy and help optimize individualized treatment methods for cervical cancer.

Ionizing radiations often lead to epigenetic changes such as DNA methylation, which alter gene expression levels without any transcriptional changes 4. The DNA methylation status of normal and malignant cells is considerably different, which indicates its biological significance in tumor development. Aberrant methylation of CpG islands in the promoter region of tumor suppressor genes is an early event in carcinogenesis, and induces malignant transformation via decreased expression and even loss of function of these genes 5. The progression of cervical cancer is dependent on multiple tumor suppressors including *PAX1*, *SOX1*, *LMX1A* and *ZNF582* 6-8.

The *PAX1* gene is located on chromosome 20p11 and consists of a paired domain (PD) and an octapeptide domain (OP). It plays an important role in the growth and development of bone, spine, thymus and parathyroid gland 9, 10. Lai et al. 11 first reported that the* PAX1* gene is significantly hypermethylated (*PAX1m*) in cervical cancer tissues compared to normal cervical tissues, and the methylation level correlated positively with the tumor grade. Subsequent studies demonstrated that the *PAX1* methylated level could accurately distinguish high-grade CIN lesions and cervical cancer. For instance, *PAX1m* can detect CIN3+ lesions with sensitivity and specificity of 86% and 85%, respectively 12, 13. In addition,* PAX1m* shows a better diagnostic performance than HPV-DNA for the triage of patients with atypical squamous cells of undetermined significance (ASCUS) 14.

Therefore, *PAX1* methylation level is a promising biomarker for cervical cancer screening and early diagnosis. However, few studies have evaluated the changes in *PAX1* gene methylation status during radiotherapy, and it is unclear whether it can predict the therapeutic response in cervical cancer. In this study, we investigated the predictive value of *PAX1* methylation status in monitoring the early response to radiotherapy in cervical cancer.

## Methods

### Patients and study design

This study was approved by the Ethics Committee of Xiangya Hospital (202010138), and all treatment procedures were carried out in accordance with the relevant guidelines. The inclusion criteria were as follows: (1) cervical cancer diagnosis by pathological biopsy and (2) no previous history of surgery and chemoradiotherapy for cervical cancer. Patients that did not complete the treatment regimen, showed recurrence, underwent non-primary treatment, or lost follow-up were excluded.

**Figure 1:**
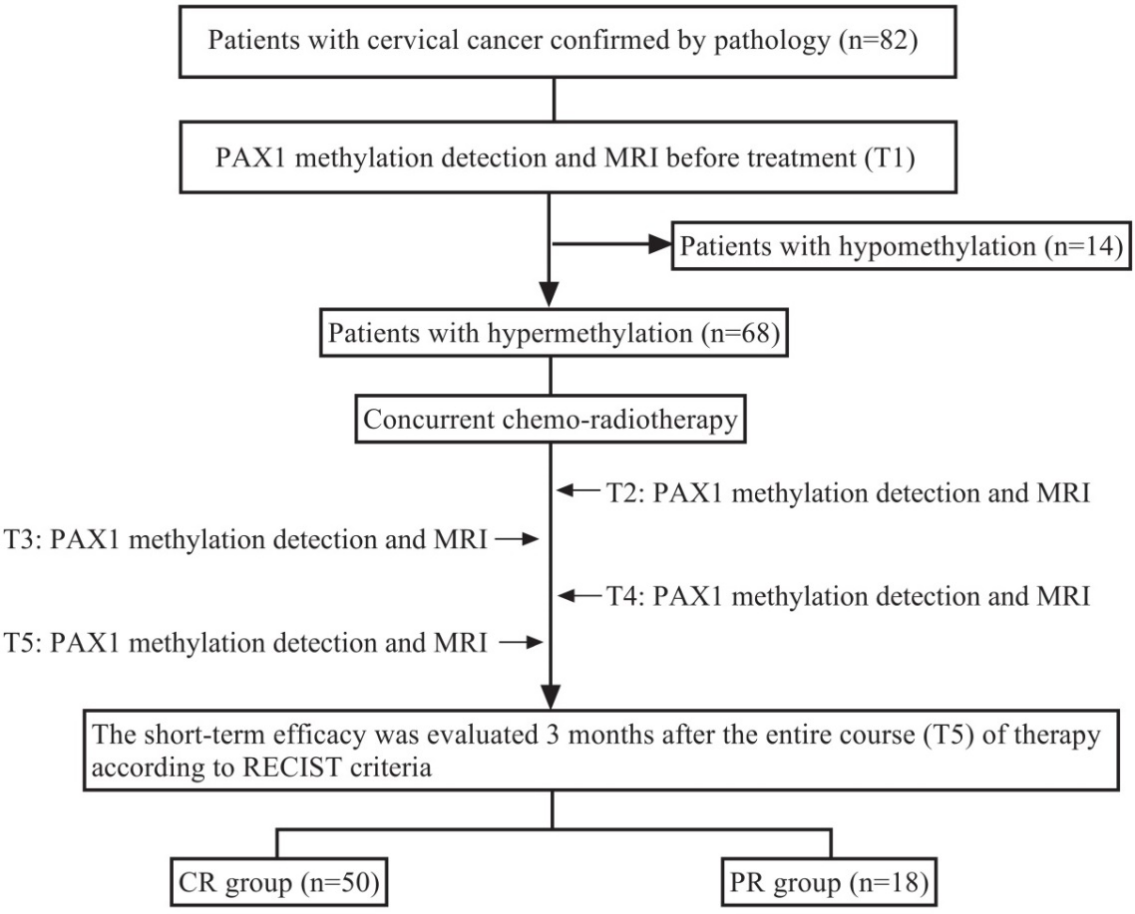
Research design. Before treatment (T1), *PAX1* was detected in patients with cervical cancer who were scheduled to receive CCRT. Hypermethylated patients were detected by MRI and methylation test at T2 (middle stage of radiotherapy), T3 (end of radiotherapy), T4 (1 month after radiotherapy), T5 (3 months after radiotherapy) time points during treatment, and were grouped with MRI.

A total of 82 cervical cancer patients were enrolled in this cohort. The patients with hypermethylation before concurrent chemo-radiotherapy (CCRT) were further monitored during radiotherapy by methylation tests and MRI at the following time points: T1 (baseline assessment before treatment), T2 (middle stage of radiotherapy), T3 (end of radiotherapy), T4 (1 month after radiotherapy) and T5 (3 months after radiotherapy). Early tumor response was determined by comparing the baseline MRI results with that of three months post-radiotherapy. The research design is illustrated in Figure 1.

### Treatment

All patients were treated with a combination of external beam radiotherapy (EBRT) and intracavitary brachytherapy (ICR). The total dose of EBRT was 45~50Gy across the entire course of treatment, and accompanied with 35~40mg/m^2^ cisplatin once a week. The treatment plan was adjusted according to the condition of the patients. ICR was initiated after 15 rounds of EBRT.

### MR imaging and evaluation

Baseline tumor assessment was performed before CCRT, and the patients were staged based on MRI and gynecological examination. The final treatment response was determined 3 months post-CCRT by MRI, visual diagnosis and physical examination, and compared to baseline. The clinical efficacy was evaluated according to RECIST criteria as complete remission (CR), partial remission (PR), stable disease (SD) or progressive disease (PD) 15. The width, height and thickness of the tumor were measured based on T2-weighted images, and tumor volume was calculated as 1/6*π*w*h*t 16. The change in tumor size (%) was calculated as (pre-treatment volume - post-treatment volume) / pre-treatment volume × 100 (%).

### Quantitative methylation-specific PCR

*PAX1* methylation was evaluated at the aforementioned time points. Cervical exfoliated cells were obtained by cervical brush during gynecological examination, centrifuged and stored in phosphate-buffered saline at -20°C. Genomic DNA was extracted using standard protocols, and converted to bisulfite form using the EZ DNA Methylation-Gold kits (Zymo Research, Irvine, CA, USA) according to the manufacturer's instructions. Methylation-specific PCR was performed on the Light Cycler LC480 system (Roche Applied Science, Penzberg, Germany) to determine the methylation level of *PAX1*. Type II collagen gene (*COL2A*) was used as an internal reference. The △Cp is the difference between the △Cp values for *PAX1m* and *COL2A*. The methylation level (△Cp) was assessed by the following formula: △Cp=Cp_target gene_ - Cp*_Col2A_* 12. The smaller △Cp value denote a higher degree of methylation detected in the collected samples. Accordingly, if △Cp≤9 (the cut-off value), *PAX1* was considered hypermethylated or positive. A decrease of △Cp value appeared indicating an increased methylation level 17. The change in △Cp value (%) was calculated using the following equation: (post-treatment △Cp - pre-treatment △Cp) / pre-treatment △Cp × 100 (%).

### Statistical analysis

SPSS software version 25.0 was used for statistical analysis. Quantitative data were expressed by means ± standard deviation (SD). Student's t test, Chi-square test or Fisher exact test was used to evaluate the relationship between *PAX1* methylation status and the clinical parameters and treatment responses. The multiple comparisons of *PAX1m* level, change of △Cp value, tumor size and tumor size regression rate between two groups at each time were performed using repeated measures analysis of variance (ANOVA) and Student's t test. Receiver operating characteristic (ROC) analysis was performed to investigate the discriminatory capacity of *PAX1* and tumor size for early response to radiotherapy. The area under the curve (AUC) was computed for the mean ROC curves. The threshold obtained from the Youden's index was used to calculate the sensitivity and specificity. All tests were performed using bilateral 95% confidence intervals (CI). *P* < 0.05 was considered statistically significant.

## Results

### Relationship between *PAX1* methylation status and clinical parameters

In this study, we performed QMSP to detect *PAX1* methylation level in 82 patients. We explored the correlation of baseline *PAX1* methylation status with clinical outcomes including age, FIGO stage, pathological type, HPV status, lymph node metastasis, tumor size and short-term efficacy. From the result, we found that the hypermethylation before CCRT was related to a superior treatment response (*P*=0.025). However, the patient age, FIGO stage, pathological type, HPV status, lymph node metastasis and tumor size were not statistically significant (*P*>0.05) (Table 1).

We also explored that 68 patients exhibiting high pre-treatment *PAX1* methylation level. We further investigated the *PAX1* methylation level for the hypermethylated group before, in the middle, immediately after, 1 month and 3 months after the CCRT. Among them, 50 patients were classified as CR and 18 patients were PR according to the 3 months post-CCRT MRI evaluation.

### Changes of* PAX1* methylation levels during treatment

We monitored the *PAX1* methylation changes of the 68 hypermethylated patients during radiotherapy. According to the MRI results, we further grouped 68 patients into CR (n=50) and PR (n=18) group. The average *PAX1* △Cp values of the CR and PR groups before radiotherapy were 5.08±1.98 and 4.32±2.00, respectively, and the △Cp of the CR group was higher than that of the PR group, not reaching significance (P=0.17). At the end of CCRT (T3), the △Cp values of the CR and PR groups increased significantly to 17.35±4.96 and 16.99±6.17, respectively (P< 0.05).

After the initiation of CCRT, the most dramatic change occurred in two groups (change rate, 183.80±163.18% vs 62.13±99.04%, P=0.00) at T2. For the CR group, △Cp values of *PAX1* methylation increased sharply from T1 to T2 and gradually thereafter. The *PAX1* △Cp values increased at T2 and T3 were significantly different from those before CCRT (P<0.05) (Table 2). In contrast, the *PAX1* △Cp values increased at a slower rate after treatment initiation in the PR group and slightly higher at T2 compared to baseline level (Table 2). A significant change in *PAX1* methylation level was observed only at the end of the treatment for the PR group. Finally, the values of △Cp was significantly less in the PR group compared to the CR group at the treatment mid-point (Table 2). Thus, results showed that the trend of △Cp value was different in patients with different therapeutic effects, especially in the middle of the stage of radiotherapy.

### Changes of tumor size during treatment

Tumor size is one of the important factors that affect efficacy. In 68 patients with high *PAX1* methylation level, we analyzed the change of tumor size (n=68) and during radiotherapy to explore the correlations between therapeutic response and the change rate of tumor size during CCRT. The mean baseline tumor size of hypermethylated patients was 23.41 ± 22.74 cm^3^ (Table 1). The mean baseline tumor size of CR and PR group were 16.08 ± 13.55 cm^3^, 43.79±30.19 cm^3^, respectively, and were remarkably decreased after radiotherapy in the two groups (P<0.05, Table 3). At each time point, tumor size in the PR group were significantly larger than those in the CR group. However, no significant difference in tumor regression rate was observed between the groups at T2 and T3 (All P<0.05, Table 3). Up to T4 and T5 time point, the tumor regression rate in CR group was significantly higher than that in the PR group. This indicated that the change trend is not obvious at the early stage of treatment.

### Predictive value of *PAX1* methylation level and tumor size in mid-treatment

**Figure 2:**
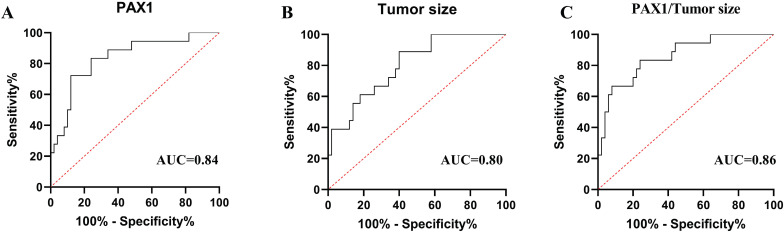
Receiver operating characteristic (ROC) curve analysis of *PAX1m* (A)*,* tumor size (B) and combined factors (C) during CCRT. The area under the curve (AUC)of each parameter's ROC curve was calculated for the predicting tumor residual group.

To evaluated and compared the diagnostic performances of *PAX1* methylation and tumor size in predicting the tumor partial response at T2 stage, we generated the ROC analysis curves (Figure 2). The AUCs of *PAX1* methylation and tumor size for predicting PR at T2 were 0.84 (P<0.05, 95% CI:0.73-0.95) and 0.80 (P<0.05, 95% CI: 0.69-0.91). The cutoff *PAX1* methylation △Cp value was 6.38 and the sensitivity, specificity, PPV and NPV for predicting PR were 0.72, 0.88, 0.68 and 0.90, respectively. Tumor size ≥ 4.59 cm³ in the middle of the treatment regimen predicted PR with the specificity of 0.60, sensitivity of 0.89, PPV of 0.44, NPV of 0.94 (Table 4).

To test the adjunct role of DNA methylation for MRI, we calculated the combined diagnostic performances of *PAX1* methylation and tumor size. As shown in Table 4, the combination of two factors at T2 had an average AUC of 0.86 (P<0.05, 95% CI:0.75-0.93), with a specificity of 0.76, a sensitivity of 0.83, PPV of 0.56, NPV of 0.93.

## Discussion

The role of *PAX1* gene detecting in cervical cancer screening and triage has been demonstrated. We analyzed the methylation level of *PAX1* gene in cervical cancer cells during different periods of CCRT using qMSP-PCR and found that *PAX1* methylation level can predict and monitor early therapeutic response.

DNA methylation is a key event in tumor genesis and progression. The tumor suppressor *PAX1* is aberrantly methylated in various human malignancies, such as oral cancer 18, esophageal cancer 19, ovarian cancer 20 etc. *PAX1* inhibits cancer cell growth by forming complexes with SETIB and WDR5 to activate phosphatase that inhibit the oncogenic MAPK and SRC pathways 21. Thus, hypermethylation of *PAX1* promoter silences its expression and promotes cancer progression. Recent studies show that *PAX1* methylation level is a reliable marker for the differential diagnosis of benign and malignant cervical growth, and has been approved in Taiwan to supplement cytological examination. Lai et al. 11 reported that the methylation rates of *PAX1* in cervical cancer were as high as 94.5%. Huang et al. showed that the level of *PAX1* methylation was associated with TNM staging in colorectal cancer 22. In present study, we detected 83% methylation rate of* PAX1* in the cervical cancer tissues. However, there were no significant differences in the age, tumor size, FIGO stage, histological types, HPV and lymphatic metastasis between the *PAX1* hypermethylated and* PAX1* hypomethylated groups, suggesting that *PAX1* methylation status may not be affected by other clinical factors.

Many researchers have previously found the influence of gene methylation status to evaluate the radiotherapy sensitivity for cervical cancer. David Guerrero-Setas et al. 23 reported that a significant correlation between *RASSF2* hypermethylation and bad prognosis of cervical cancer. Wu et al. 24 demonstrated that *ZNF582* negative could increase resistance to radiation and affect the prognosis. Meanwhile, Ph Su et al. 21 analyzed DNA methylation from TCGA database that disease-free survival (PFS) and overall survival (OS) of patients with the hypermethylated *PAX1* gene was significantly shorter than the hypomethylated patients in uterine cervical cancer. Interestingly, our data show that *PAX1* gene hyper-methylation was associated with an excellent therapeutic response, lower methylation level or negative methylation indicates partial response. The explanation might be that therapy difference might be the main reason for this result because our patients underwent CCRT compared with other studies that involved patients underwent surgery or neoadjuvant chemotherapy. Therefore, we infer that the *PAX1* hypomethylation may be insensitive to radiation, which may lead to a poor result.

DNA methylation status is reversible and can be altered in response to external stimuli. Antwih et al.^25^ found that ionizing radiations significantly altered the level of gene methylation by downregulating *DNMT2* in breast cancer cells. Wu et al.^24^ reported that *ZNF582* methylation levels decreased in patients receiving neoadjuvant radiotherapy. However, few studies have reported the functional of *PAX1* methylation during CCRT. In our study as well, *PAX1* △Cp value increased after treatment and stabilized after radiotherapy, indicating that *PAX1* methylation levels decreased steadily during CCRT. The gradual decrease in *PAX1* methylation level was consistent with the change in tumor size in radiotherapy. Effective anticancer therapy leads to the destruction of tumor cell membrane integrity and tumor dissolution, thus reducing the density of tumor size. The decreased methylation levels may suggest fewer cancer cell after treatment. Therefore, the fluctuation of the methylation level is not obvious after CCRT, which may be related to the difficulty in obtaining effective cells. According to our results, *PAX1* methylation levels decreased significantly by the mid-point of CCRT in the CR but not PR groups.

Tumor size is closely related to the prognosis of cervical cancer patients 26-28_._ However, in our study, the mid-RT tumor regression rates were similar between the CR and PR group, and significant differences were seen only after the end of treatment. The unsatisfactory outcome of short-term treatment may be lagging at the early reflection. Furthermore, changes in tumor size varied only at the end of radiotherapy. These data indicate that changes in *PAX1* methylation levels precede tumor size because epigenetic changes in tumor cell may have occurred in the early stages of treatment. Therefore, monitoring the dynamic change in *PAX1* methylation can help optimize individualized therapeutic strategies. Early decrease of *PAX1* level indicates a better response to radiotherapy and a favorable prognosis in cervical cancer, whereas minute fluctuations may reflect a therapeutically unresponsive tumor.

In present study, *PAX1* methylation level showed slightly better performance than tumor size for distinguishing between CR or residual tumor cells (AUC 0.84 vs 0.80). In addition, at the △Cp threshold of 6.38, *PAX1* methylation distinguished CR and PR with the high specificity of 0.88 compared to only 0.60 for tumor size. Therefore, the reasonable sensitivity (0.72 vs 0.89) and high specificity (0.88 vs 0.60) of* PAX1* methylation make it a promising predictive marker of the short-term efficacy of CCRT in cervical cancer, and combining it with MRI may further enhance the accuracy of therapeutic monitoring. Radiotherapy significantly improves the treatment outcomes in cervical cancer in a dose-dependent manner. It is critical to rapidly evaluate the early treatment response, precisely and sensitively, to adjust the treatment plan and reduce the risk of adverse reactions. Taken in aggregate, *PAX1* methylation level is a better indicator of the early molecular changes in the tumor during treatment than MRI parameters to timely detect patients who are not sensitive to radiotherapy and adjust treatment regimen. In addition, considering the second pathological biopsy may bring trauma and subjective pain to patients because the tumor shrinks after treatment, so cytology has the advantage of more straightforward, convenient and non-invasive compared pathological examination when needed to collect samples at multiple time points. Methylation PCR can be easily performed using cervical exfoliated cells and does not require cervical tissue.

This study has several limitations that ought to be considered. First, the small sample size limits the generalizability of the results, although each patient underwent five tests that increased the data pool. Nevertheless, our findings need to be validated on larger cohorts. Second, we only analyzed the effect of *PAX1* methylation on short-term therapeutic efficacy, and its effect on long-term survival will need to be confirmed by long-term follow-up. Third, in the absence of histological confirmation, it may be impractical to judge through magnetic resonance imaging, and future animal experiments will help us to understand the early tumor response better.

In conclusion, we found that *PAX1* gene methylation status will change under the influence of radiation, and may predict early treatment response of cervical cancer patients post-radiotherapy. Our result indicates that *PAX1* methylation as a promising biomarker plays an important role in monitoring and treatment following up of cervical cancer. Therefore, by combining methylation detection with traditional imaging methods, it may provide a new method for monitoring the effect of treatment.

## Figures and Tables

**Table 1:** Relationship between *PAX1* promoter methylation and clinical parameters.

Characteristics		*PAX1* hypo-(n=14)	*PAX1* hyper-(n=68)	*P*
Age (years)		57.21±6.267	54.77±8.75	0.32^#^
FIGO stage				0.62^Δ^
	<ⅡB	2(14.29)	6(8.82)	
	≥ⅡB	12(85.71)	62(91.18)	
Pathology				0.07^Δ^
	SCC	12(85.71)	67(98.53)	
	AC	2(14.29)	1(1.47)	
Lymph node metastasis				0.16^*^
	No	6(42.86)	43(63.24)	
	Yes	8(57.14)	25(36.76)	
HPV				0.17^*^
	Negative	7(50.00)	21(30.88)	
	Positive	7(50.00)	47(69.12)	
Size of tumor (cm^3^)		21.50±24.15	23.41±22.74	0.78^#^
Tumor response				0.025^*^
	CR	6	50	
	PR	8	18	

Note: Data presented as mean ± standard deviation or n (%). SCC: squamous cell carcinoma; AC: adenocarcinoma; FIGO: International Federation of Gynecology and Obstetrics; HPV: human papilloma virus. CR: complete response; PR: partial response. ^#^T test; *Chi-square test; ^Δ^Fisher's test.

**Table 2:** Difference of *PAX1* methylation level between CR and PR groups at each time points of treatment for cervical cancer.

Time points	△Cp			△Cp change rate (%)		
	CR (n=50)	PR (n=18)	*P*	CR (n=50)	PR (n=18)	*P*
T1	5.08±1.98	4.32±2.00	0.17	-	-	-
T2	12.57±5.88	6.15±4.09	**6.50×10^-5^**	183.80±163.18	62.13±99.04	**0.004**
T3	17.35±4.96	16.99±6.17	0.80	315.82±258.21	381.19±194.96	0.38
T4	15.60±4.76	14.88±4.94	0.59	274.82±220.35	302.69±200.91	0.64
T5	16.66±4.95	17.09±5.67	0.76	300.49±235.44	377.33±280.21	0.26

Note: T1 (pre-radiotherapy), T2 (middle stage of radiotherapy), T3 (end of radiotherapy), T4 (1 month after radiotherapy) and T5 (3 months after radiotherapy). CR: complete response; PR: partial response.

**Table 3:** Comparison of change rate of tumor size during radiotherapy between CR and PR groups.

Time points	Tumor size (cm^3^)			Tumor size regression (%)		
	CR (n=50)	PR (n=18)	*P*	CR (n=50)	PR (n=18)	*P*
T1	16.08±13.55	43.79±30.19	**2.00×10^-6^**	-	-	-
T2	5.00±4.74	12.73±8.40	**1.10×10^-5^**	65.16-25.27	63.82-20.94	0.83
T3	1.05±1.45	3.73±2.43	**5.51×10^-7^**	92.25-10.34	87.51-13.99	0.14
T4	0.23±0.53	1.83±1.56	**2.05×10^-8^**	98.30-4.34	94.28-5.57	**3.20×10^-5^**
T5	0.00±0.00	0.77±0.71	**7.72×10^-11^**	100.00-0.00	97.67-2.51	**5.83×10^-22^**

Note: T1 (pre-radiotherapy), T2 (middle stage of radiotherapy), T3 (end of radiotherapy), T4 (1 month after radiotherapy) and T5 (3 months after radiotherapy).CR: complete response; PR: partial response.

**Table 4:** The performance of two detection methods at the middle stage of treatment.

Variable	Cut-off	AUC	Sensitivity	Specificity	PPV	NPV	95%CI	*P* value
*PAX1*	6.38	0.84	0.72	0.88	0.68	0.90	0.73-0.95	**2.50×10^-5^**
Tumor size (cm^3^)	4.59	0.80	0.89	0.60	0.44	0.94	0.69-0.91	**1.65×10^-4^**
Combined factors	-	0.86	0.83	0.76	0.56	0.93	0.75-0.93	**7.00×10^-6^**
